# Teaching an old pET new tricks: tuning of inclusion body formation and properties by a mixed feed system in *E*. *coli*

**DOI:** 10.1007/s00253-017-8641-6

**Published:** 2017-11-20

**Authors:** David J. Wurm, Julian Quehenberger, Julia Mildner, Britta Eggenreich, Christoph Slouka, Andreas Schwaighofer, Karin Wieland, Bernhard Lendl, Vignesh Rajamanickam, Christoph Herwig, Oliver Spadiut

**Affiliations:** 1 Research Division Biochemical Engineering, Institute of Chemical, Environmental and Biological Engineering, TU Wien, Vienna, Austria; 2 Christian Doppler Laboratory for Mechanistic and Physiological Methods for Improved Bioprocesses, Institute of Chemical, Environmental and Biological Engineering, TU Wien, Vienna, Austria; 3 Institute of Chemical Technologies and Analytics, TU Wien, Vienna, Austria

**Keywords:** *Escherichia coli* BL21(DE3), pET expression system, Lactose, Inclusion body properties, Inclusion body size, Inclusion body purity

## Abstract

Against the outdated belief that inclusion bodies (IBs) in *Escherichia coli* are only inactive aggregates of misfolded protein, and thus should be avoided during recombinant protein production, numerous biopharmaceutically important proteins are currently produced as IBs. To obtain correctly folded, soluble product, IBs have to be processed, namely, harvested, solubilized, and refolded. Several years ago, it was discovered that, depending on cultivation conditions and protein properties, IBs contain partially correctly folded protein structures, which makes IB processing more efficient. Here, we present a method of tailored induction of recombinant protein production in *E*. *coli* by a mixed feed system using glucose and lactose and its impact on IB formation. Our method allows tuning of IB amount, IB size, size distribution, and purity, which does not only facilitate IB processing, but is also crucial for potential direct applications of IBs as nanomaterials and biomaterials in regenerative medicine.

**Electronic supplementary material:**

The online version of this article (10.1007/s00253-017-8641-6) contains supplementary material, which is available to authorized users.

## Introduction

*Escherichia coli* is the most widely used host organism for recombinant protein production due to its well-studied genome, the existence of numerous cloning vectors and engineered strains, as well as the possibility of cheap and straight-forward cultivation to high cell densities yielding high product titers (Choi et al. 2006; Huang et al. 2012; Joseph et al. 2015; Liu et al. 2015). As generally known, a careful balance between transcription and protein folding must be realized to increase the amount of soluble product (SP) in *E*. *coli*. If the folding machinery gets overwhelmed, correctly folded secondary structures cannot be formed and inclusion bodies (IBs) are produced (e.g., (Gatti-Lafranconi et al. 2011; Marschall et al. 2016)). In this respect, induction temperature, pH of the cultivation medium, and changes in the amino acid sequence of the product have a profound effect (Strandberg and Enfors 1991).

The by far most used *E*. *coli* strain is *E*. *coli* BL21(DE3) as it is known for a reduced amount of proteases and prevented plasmid loss (Jia and Jeon 2016; Liu et al. 2015; Rosano and Ceccarelli 2014). This strain is mostly used in combination with the T7-based pET expression system, which is usually induced by isopropyl-β-d-thiogalactopyranoside (IPTG), a nonmetabolizable molecular mimic of allolactose, known for strong induction (Bashir et al. 2016; Durani et al. 2012; Jia and Jeon 2016; Marbach and Bettenbrock 2012; Rosano and Ceccarelli 2014; Wurm et al. 2016). However, IPTG puts a high metabolic burden on *E*. *coli* (Dvorak et al. 2015; Haddadin and Harcum 2005), and thus causes the enhanced formation of IBs (Sina et al. 2015; Zhang et al. 2015). For a long time, IBs were considered to be aggregates of misfolded and inactive product, which is why IB formation was highly undesired for decades (Baneyx 1999; Choi et al. 2006; Marston 1986). However, in the past few years, IBs were found to have many advantages, such as significantly higher primary yields, simple separation from cell matter, high purity, and resistance to proteolysis (Choi et al. 2006; Ramon et al. 2014; Yamaguchi and Miyazaki 2014). Consequently, marketed biopharmaceuticals from *E*. *coli*, such as hormones, growth factors, interleukins, and insulin, are nowadays mostly produced as IBs, followed by solubilization and refolding to get soluble target product (Eiberle and Jungbauer 2010; Schmidt 2004; Yamaguchi and Miyazaki 2014).

Furthermore, it was found that, depending on cultivation conditions, IBs contain correctly folded secondary structures (Gatti-Lafranconi et al. 2011). The presence of such structures actually allows a comparably mild treatment during IB processing to maintain the already correctly folded secondary structures and thus increase the refolding yield. Different products, such as granulocyte-colony stimulating factor, truncated forms of tumor necrosis factor, lymphotoxin α, and the marker protein green fluorescent protein, have already been successfully produced by that strategy (Jevsevar et al. 2005; Peternel et al. 2008a, b; Singh et al. 2015b; Villaverde et al. 2015).

However, most of the current recombinant protein production processes with *E*. *coli* still aim at the production of SP instead of IBs. In this respect, several approaches for tuning recombinant protein expression in BL21(DE3) and thus, the level of SP and IB have been proposed. While many studies suggest suboptimal growth conditions to slow down all cellular processes, including transcription and translation (Peternel and Komel 2011; Vera et al. 2007), others propose supplying limiting amounts of IPTG (below 1 μmol IPTG/g biomass) to tune down transcription (Striedner et al. 2003). In this respect, we used lactose as inducer instead of IPTG in previous studies (Wurm et al. 2017; Wurm et al. 2016), as it enhances correct protein folding and increases cell fitness (Bashir et al. 2016; Fruchtl et al. 2015; Ma et al. 2013; Wurm et al. 2016). We demonstrated that actually both SP titer and IB titer were influenced by the specific uptake rate of lactose (*q*_s,lac_), which in turn depends on the specific uptake rate of glucose (*q*_s,glu_; (Wurm et al. 2016, 2017)). We generated a mechanistic model (Wurm et al. 2016, 2017) for this delicate balance between ATP-related uptake of lactose at low *q*_s,glu_ (Johnson and Brooker 2004; Kaback 2015) and carbon catabolite repression at high *q*_s,glu_ ((Bruckner and Titgemeyer 2002; Kremling et al. 2015; Warner and Lolkema 2003); Supplementary Fig. S1).

In this follow-up study, we investigated the correlation between *q*_s,lac_ and IB formation in more detail. For this purpose, we decoupled growth and induction by keeping *q*_s,glu_ constant and applying different *q*_s,lac_ to potentially vary IB titer and properties. Motivated by a study of Peternel et al., who showed that IB properties strongly depend on cultivation conditions (Peternel et al. 2008b), we hypothesized that not only the amount of IBs, but also IB properties can be tuned by adjusting different *q*_s,lac_ and thus different levels of induction. Furthermore, we analyzed the effects of these conditions on the expression of SP to retrieve information about the total expression capacity of *E*. *coli*. For this purpose, we used the model protein enhanced green fluorescence protein (eGFP), which is a representative of the beta-barrel protein class and prominent for protein quality studies.

## Materials and methods

### Strain

For all experiments, *E*. *coli* BL21(DE3) (Life technologies, Carlsbad, CA, USA), transformed with a pET21a(+) vector carrying the gene coding for enhanced green fluorescent protein (eGFP) was used as expression host.

### Bioreactor cultivations

All fermentations comprised a batch cultivation followed by an uninduced fed-batch for biomass generation and a 12 h induction phase. Experiments were carried out in DASbox® Mini Bioreactors (Eppendorf, Hamburg, Germany) with a working volume of 250 mL. CO_2_ and O_2_ in the off-gas were analyzed by a DASGIP® GA gas analyzer (Eppendorf, Hamburg, Germany); pH by a pH-Sensor EasyFerm Plus (Hamilton, Reno, NV, USA); and dissolved oxygen (dO_2_) by a Visiferm DO 120 electrode (Hamilton, Reno, NV, USA). dO_2_ was kept above 40% oxygen saturation throughout the whole fermentation by supplying 2 vvm of a mixture of pressurized air and pure oxygen. Biomass concentration was estimated using a soft-sensor-tool (Wechselberger et al. 2013), feed-flowrates were adjusted with the DASbox® MP8 Multi Pump Module, pH was kept at 7.2 by supplying 12.5% NH_4_OH, stirring speed was set to 2000 rpm, and temperature was set to 35 °C during batch and fed-batch and was lowered to 30 °C during induction. All process parameters were logged and controlled by the DASware® control.

**Fig. 1:**
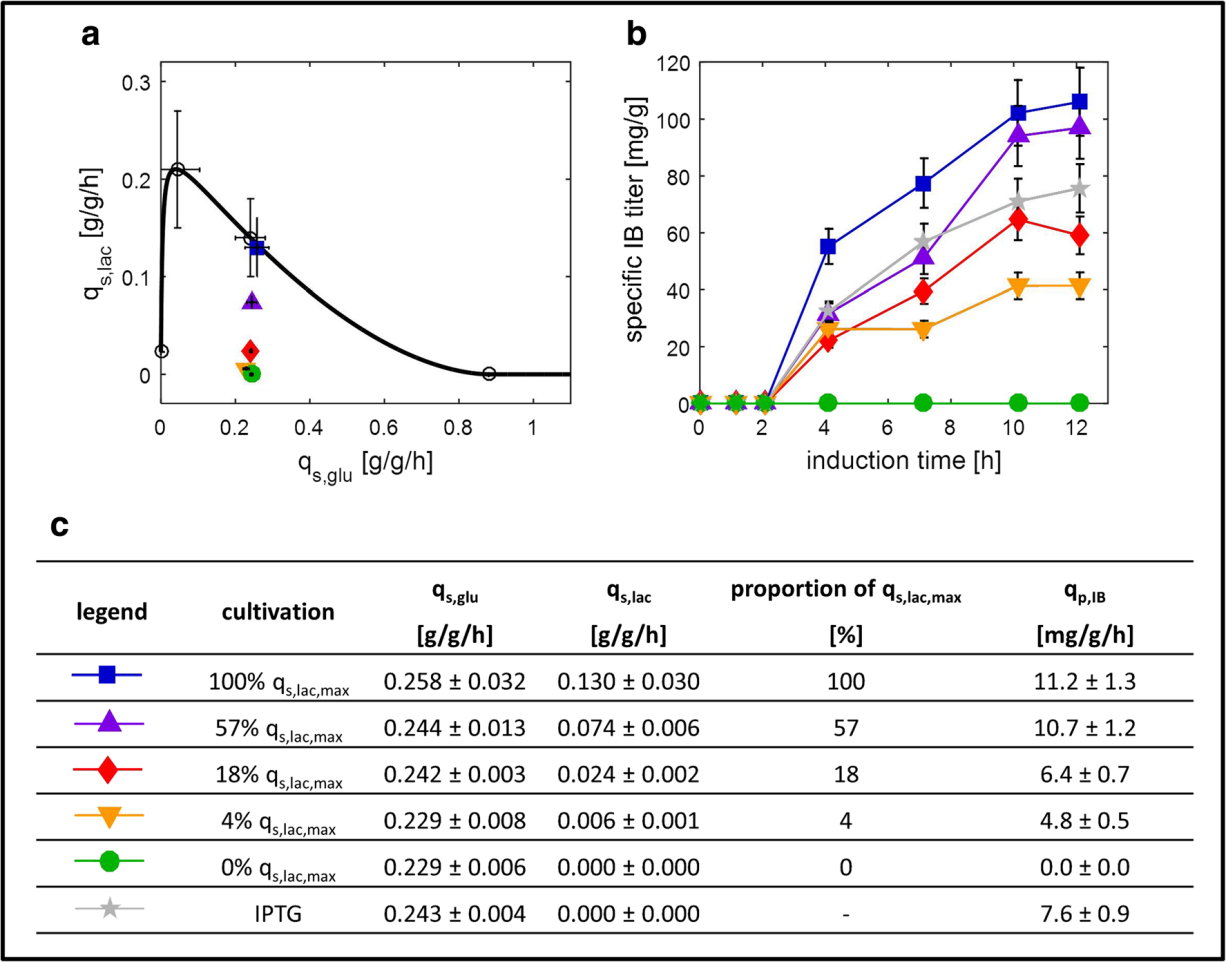
Tuning IB formation rate. **a** Black line indicates the maximum specific uptake rate of lactose (*q*_s,lac_) as a function of the specific uptake rate of glucose (*q*_s,glu_) for an *Escherichia coli* BL21(DE3) strain producing enhanced green fluorescent protein (eGFP). Data points (open circles) were obtained from several batch and fed-batch cultivations and fitted by the mechanistic model according to our previous study (Wurm et al. 2016, 2017). Colored symbols indicate performed experiments as shown in (**b**) and (**c**). Error bars indicate deviation of the respective *q*_s_ over induction time. **b** Specific IB titer in mg_eGFP_/g_cells_ as a function of time for lactose and IPTG (0.5 mM) induction. **c** Summary of specific sugar uptake rates (*q*_s_) and specific IB formation rates (*q*_p,IB_). The error bars of the specific IB titers indicate the standard deviation (namely 11.25%), which was identified by performing biological replicates of the center point (i.e., 18% *q*_s,lac,max_)

Five hundred milliliters of sterile DeLisa pre-culture medium (DeLisa et al. 1999) supplemented with 0.1 g/L ampicillin and 8 g/L glucose were aseptically inoculated from frozen stocks (1.5 mL, − 80 °C). Pre-cultures were grown overnight (20 h) in 2500-mL high-yield shake flasks in an Infors HR Multitronshaker (Infors, Bottmingen, Switzerland) at 37 °C and 250 rpm. One hundred-fifty milliliters of DeLisa batch medium (DeLisa et al. 1999) supplemented with 0.1 g/L ampicillin and 10 g/L glucose were inoculated with 15 mL of pre-culture. After sugar depletion, a fed-batch phase to reach about 25 g_cells_/L using a glucose feed with 250 g/L glucose was carried out. Induction was performed by addition of 0.5 mM IPTG or supplementing the feed with different amounts of lactose to reach the *q*_s,glu_ and *q*_s,lac_ values displayed in Fig. 1c.

#### Sampling

Samples were taken at the beginning and end of the batch and fed-batch phase, furthermore during the induction phase after 0, 1, 2, 4, 7, 10 and 12 h. Quantification of biomass dry cell weight (DCW) was performed gravimetrically as in (Wurm et al. 2016); substrates and metabolites were measured by high-pressure liquid chromatography (HPLC) as in (Wurm et al. 2016).

#### Product analysis

##### Product titer quantification by reversed-phase HPLC

Cell pellets of 5 mL fermentation broth were resuspended (100 mM Tris, 10 mM EDTA pH 7.4) to 4.0 g/L DCW and homogenized at 1500 bar for six passages (EmulsiflexC3; Avestin, Ottawa, Canada). After centrifugation (15 min, 13,000 rcf, 4 °C), the supernatant was used for analysis of SP. For IB quantification, the pellet was washed twice [(i) 50 mM Tris, 5 mM EDTA, pH 8.0; (ii) 50 mM Tris, 0.5 M NaCl, 0.02% (*w*/*v*) Tween 80, pH 8], aliquoted and stored at − 20 °C. Pellets were resuspended in a solution containing 1 part Tris-buffer (50 mM Tris, 5 mM EDTA, pH 8.0) and four parts solubilization buffer (6 M guanidine hydrochloride (GuHCl), 50 mM Tris, pH 8.0 with 5.0% (*v*/*v*) 2-mercaptoethanol added right before use), incubated for 2 h on a shaker at room temperature and vortexed every 30 min. Product quantification was carried out by HPLC analysis (UltiMate 3000; Thermo Fisher, Waltham, MA, USA) using a reversed phase column (EC 150/4.6 Nucleosil 300-5 C8; Macherey-Nagel, Düren, Germany). The product was quantified with an UV detector (Thermo Fisher, Waltham, MA, USA) at 280 nm using bovine serum albumin as standard. Mobile phase was composed of water (buffer A) and acetonitrile (buffer B) both supplemented with 0.1% (*v*/*v*) tetrafluoride acetic acid. A linear gradient from 30% (*v*/*v*) acetonitrile to 100% acetonitrile was applied. The error bars in all figures displaying product titers were identified by performing biological replicates of the center point (18% *q*_s,lac,max_) and was quantified to be 11.25% for IBs and 11.50% for SP.

##### Size determination by scanning electron microscopy (SEM)

Washed and aliquoted IB samples were resuspended in ultrapure water. One hundred microliters of appropriate dilution of the suspension were pipetted on a gold-sputtered (10–50 nm) polycarbonate filter (Millipore-Merck, Darmstadt, Germany) using reusable syringe filter holders with a diameter of 13 mm (Sartorius, Goettingen, Germany). One hundred microliters of ultrapure water were added and pressurized air was used for subsequent filtration. Additional 200 μL of ultrapure water were used for washing. The wet filters were fixed on a SEM sample holder using graphite adhesive tape and subsequently sputtered with gold to increase the contrast of the sample. SEM was performed using a QUANTA FEI SEM (Thermo Fisher, Waltham, MA, USA) using a secondary electron detector (SED). The acceleration voltage of the electron beam was set between 3 to 5 kV. The diameters of the IBs were evaluated by measuring 50 IBs on SEM pictures using the ImageJ plugin Fiji (Laboratory for Optical and Computational Instrumentation (LOCI), University of Wisconsin-Madison, USA).

##### Morphology analysis by atomic force microscopy (AFM)

For determination of morphological aspects of IBs, samples were prepared the same way as for SEM except for gold sputtering, which was not necessary for these measurements. Measurements were performed on a WITec alpha 300RSA+ (WITec GmbH, Ulm, Germany) in tapping mode (AC).

##### Secondary structure analysis by infrared spectroscopy (IR)

IR measurements were performed by an external-cavity quantum cascade laser-based IR transmission setup using the path length of 38 μm, described in detail by Alcaraz et al. (Alcaraz et al. 2015). Calculation of degree of spectral overlap by *s*_1,2_ has been described by Schwaighofer et al. (Schwaighofer et al. 2016).

### Solubilization and refolding of IB

Homogenized cell pellets were resuspended in ultrapure water and 30 μL of the suspension were pipetted into 96 microtiter plates. Subsequently, 70 μL of urea stock solution supplemented with 50 mM Tris at pH 8 were simultaneously added to each well (Qi et al. 2015).

Refolding was carried out at 30 °C for 4.5 h by diluting 10 μL of solubilizate with 190 μL of refolding buffer (50 mM Tris, 100 mM NaCl, 1 mM DTT, pH 7.5) (Enoki et al. 2004) resulting in a final protein concentration of 0.2 mg/mL.

### Impurity monitoring

Impurity monitoring to asses purity after solubilization and refolding was carried out chromatographically (UltiMate 3000; Thermo Fisher, Waltham, MA, USA) using a high-performance size-exclusion chromatography column (MAbPac™ SEC-1, Thermo Scientific, Waltham, MA, USA). For solubilized samples a GuHCl buffer (4 M GuHCl, 50 mM Bis-Tris, 300 mM NaCl, pH 6.8) and for refolded samples a phosphate buffer (100 mM Na_2_HPO_4_, 300 mM NaCl, pH 6.8) were used as mobile phase. The flowrate was kept constant at 0.2 mL/min, the column oven temperature was 25 °C, and the method lasted 17 min. An exemplary chromatogram is displayed in Supplementary Fig. S2.

## Results

### Product titer

To potentially tune the titer of eGFP, we adjusted four different *q*_s,lac_ at a *q*_s,glu_ of around 0.25 g/g/h (Fig. 1a, c), which allows both cell growth and increased recombinant product formation (Wurm et al. 2016). Additionally, we performed a control experiment without induction to rule out effects of basal expression, as the pET system is described to be leaky (Huang et al. 2012; Jia and Jeon 2016), as well as an experiment where we induced with the standard inducer IPTG at a concentration of 0.5 mM (Bashir et al. 2016; Durani et al. 2012; Jia and Jeon 2016; Marbach and Bettenbrock 2012; Rosano and Ceccarelli 2014; Wurm et al. 2016). To assure reproducibility, we performed a biological replicate of the center point (i.e., 18% *q*_s,lac,max_). The biomass concentration during induction of all cultivations can be found in the Supplementary Fig. S3.

#### IB titer

Figure 1b presents the specific IB titer, measured by reversed phase chromatography, as a function of time for 12 h of induction. Throughout the entire induction, there was a clear correlation between the specific IB formation rate (*q*_p,IB_) and *q*_s,lac_, namely, the higher *q*_s,lac_ and the higher *q*_p,IB_, leading to final titers which varied by a factor of nearly three after 12 h of induction (40 vs. 110 mg_eGFPIB_/g_cells_; Fig. 1b). Interestingly, we obtained a higher specific IB titer when we adjusted *q*_s,lac_ at 100% *q*_s,lac,max_ and 57% *q*_s,lac,max_, respectively, compared to induction with 0.5 mM IPTG (Fig. 1b, c), emphasizing the power of lactose as a nontoxic and cheap inducer. Surprisingly, we found IBs only after more than 2 h of induction (Fig. 1b). Since we detected soluble eGFP right after induction (Supplementary Table S1), we speculate that the amount and the size of IBs in the first 2 h of induction were below the detection limit of the applied analytics.

#### Soluble and total product titer

Even though the main focus of this study was the investigation of IBs, we also analyzed SP and total product titers. With respect to SP, we observed the same correlation between *q*_s,lac_ and *q*_p_ as seen for IBs during the first 4 h of induction, namely, the higher *q*_s,lac_ and the higher *q*_p,SP_. However, after 12 h of induction, the highest specific SP titer was obtained at the lowest *q*_s,lac_. Apparently, cells which were strongly induced right from the beginning of induction somehow reduced *q*_p,SP_ after a certain time, whereas cells induced at a low *q*_s,lac_ of only 4% *q*_s,lac,max_ steadily produced SP over time (Supplementary Table S1).

The total productivity also showed a clear trend in the first 4 h of induction, as increasing *q*_s,lac_ gave more total product (Supplementary Table S1). However, after 12 h of induction, all induction conditions resulted in comparable amounts of total product.

Summarizing, with respect to product titer, we concluded that, (1) *q*_p,IB_ can be tuned by *q*_s,lac_ over the whole induction time; (2) in the first 4 h of induction, higher *q*_s,lac_ gave higher *q*_p,SP_, while after 12 h of induction, this situation was reversed; and (3) after 12 h of induction, the amount of total product was comparable for all induction conditions tested.

### Tuning IB properties

In order to potentially link IB properties to induction conditions, we analyzed size, morphology, size distribution, and the presence of secondary structures of the formed IBs.

#### IB size

**Fig. 2:**
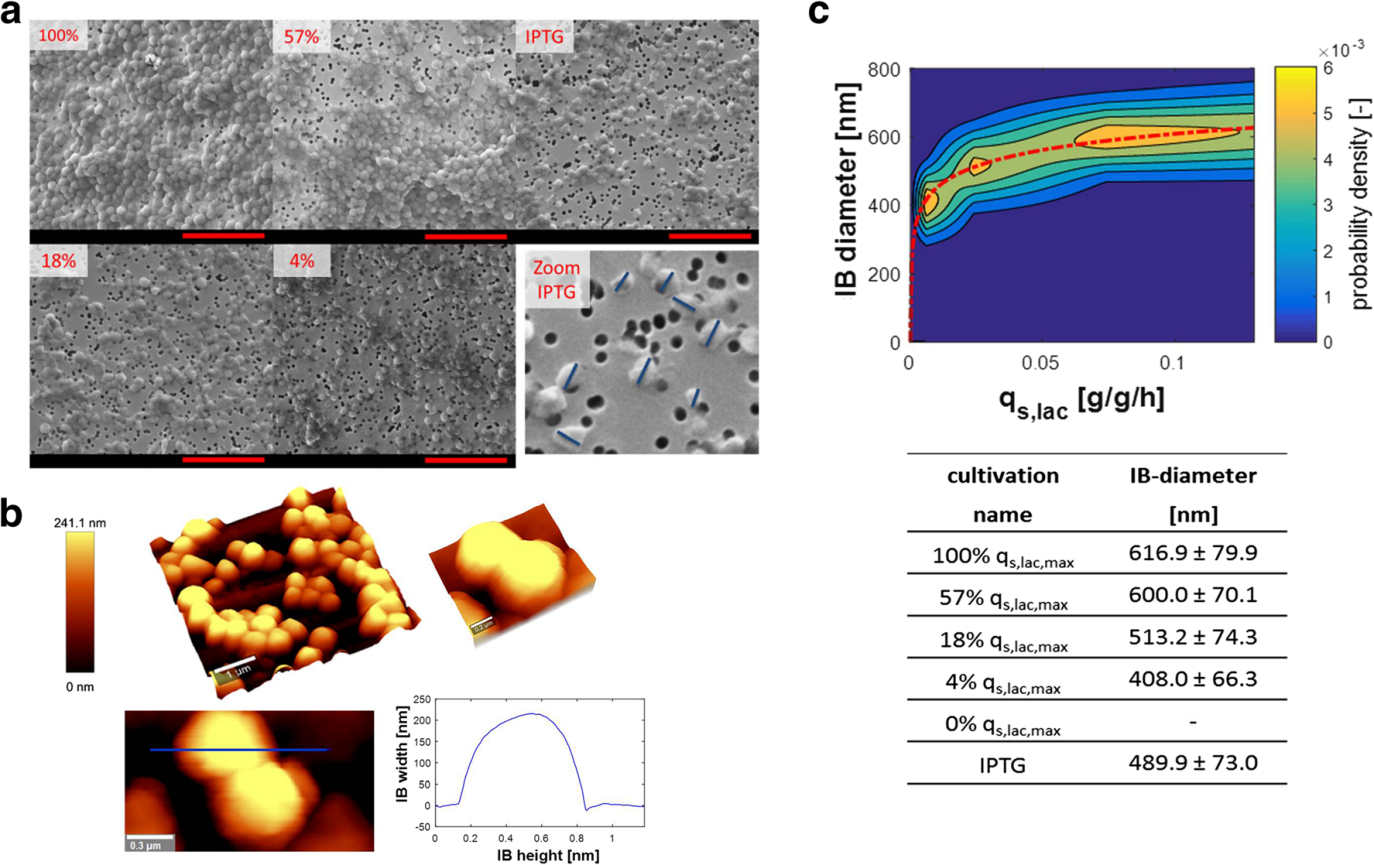
Tuning the size of IBs. **a** Scanning electron microscopy pictures of IBs from different cultivations used to asses IB size, as exemplarily shown in lower right figure. Percentage indicates proportion of maximum specific lactose uptake rate (*q*_s,lac_) used for induction. Red scale bars: 5 μm. 3.5-fold zoom for IPTG induction (lower right). **b** (i) exemplary atomic force microscopy picture of typical IBs showing spherical shape, (ii) and (iii) zoom in on IB particle, and (iv) topography cross-section of an isolated IB (indicated as a blue line in iii). **c** Probability density plot of IB size distribution after 12 h of induction as a function of *q*_s,lac_ showing that IB size can be tuned by *q*_s,lac_. Red-dashed line indicates logarithmic fit between IB size and *q*_s,lac_ (degree of freedom = 2, *R* ^2^ = 0.99). IB diameter with standard deviation from different cultivations after 12 h of induction are shown in the table. Standard deviation was evaluated from measuring 50 IBs per sample

We assessed IB size by scanning electron microscopy (SEM; Fig. 2a), supported the results by atomic force microscopy (AFM; Fig. 2b) and correlated the IB size to the respective q_s,lac_ (Fig. 2c). In fact, we were able to tune IB size by induction, as shown in Fig. 2c. A clear correlation between *q*_s,lac_ and IB size was observed: smaller IBs were produced when less lactose was specifically taken up (logarithmic fit, degree of freedom = 2, *R* ^2^ = 0.991).

#### IB morphology

Using AFM analysis, we found that eGFP IBs were of spherical shape (exemplarily shown in Fig. 2b), whose surface area can be calculated by *A* = *d* ^2^ · *π*. This underlines the high importance of the IB diameter (*d*) as it impacts the surface area (*A*), by the power of 2. Thus, it is advantageous to produce large IBs in order to minimize the surface area, where impurities can potentially adhere to.

#### IB size distribution as a function of time

**Fig. 3:**
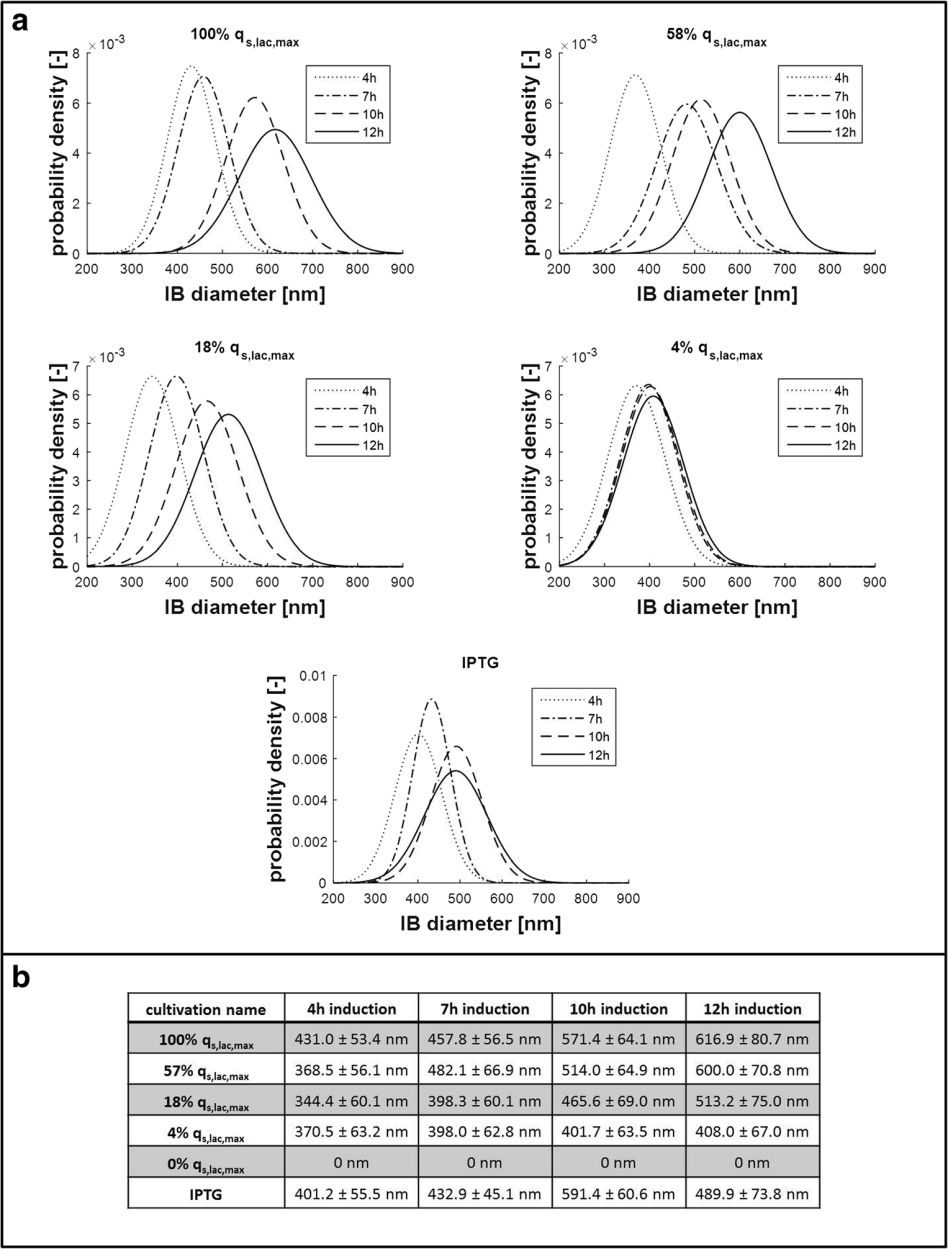
**S**ize distribution of IBs over induction time. **a** Probability density plot of IB size distribution as a function of induction time indicating that IB size increases, while also, the distribution gets broader over time for different induction conditions. **b** IB diameter with standard deviation from different cultivations conditions at different time points of induction

We found that not only IB size, but also IB size distribution increased as a function of induction time (Fig. 3). Although this trend was not as apparent for induction by IPTG, we observed an increasingly broad size distribution of formed IBs for all experiments with lactose induction (100% *q*_s,lac_, 57% *q*_s,lac,_ 18% *q*_s,lac_, and 4% *q*_s,lac_). We explain this phenomenon by the generally accepted hypothesis that the IB is passed on to only one daughter cell after cell division, leaving one daughter cell without IB and one daughter cell with an IB that continues to grow (Peternel and Komel 2011). Thus, in order to get an IB population of distinct size, which is not only important for IB processing, but also for potential direct application as nanomaterials and biomaterials (Diez-Gil et al. 2010; Garcia-Fruitos et al. 2009; Garcia-Fruitos et al. 2012; Peternel and Komel 2011; Upadhyay et al. 2012; Villaverde et al. 2015), we recommend short induction times.

#### IB secondary structures

**Fig. 4:**
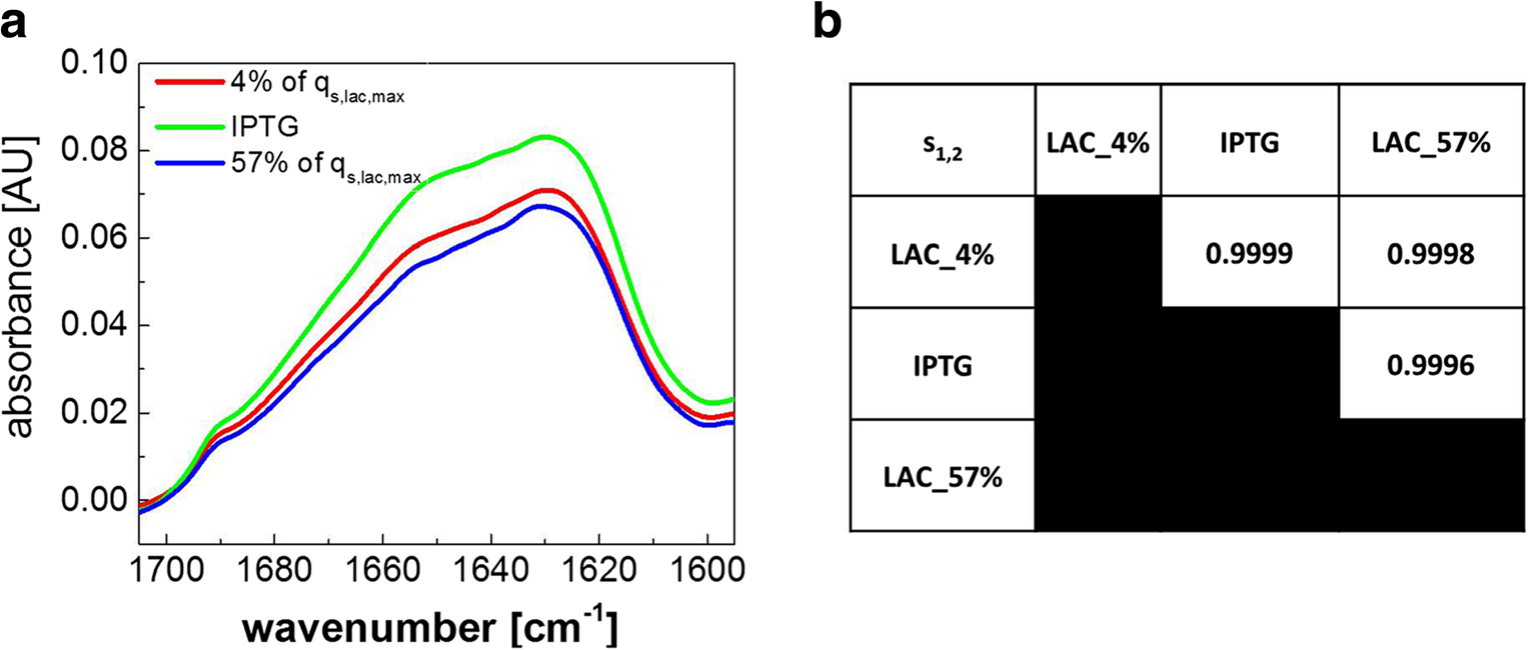
Secondary structure of IBs measured by infrared (IR) spectroscopy. **a** IR spectra of IBs from different induction regimes. Maxima for β-sheet secondary structure appear at approx. 1630 and 1690 cm^−1^ in the IR spectrum, whereas the shoulder at approx. 1655 cm^−1^ is attributed to α-helical secondary structure. **b** Table shows degree of spectral overlap (*s*_1,2_) for IBs from different induction regimes (4% *q*_s,lac,max_ (small IBs, Ø = 408 nm); IPTG (medium IBs, Ø = 490 nm); and 57% *q*_s,lac,max_ (large IBs, Ø = 600 nm)) calculated according to Schwaighofer et al. (Schwaighofer et al. 2016) demonstrating a very high degree of spectral overlap for all samples. The value of *s*_1,2_ ranges from 0 to 1, corresponding to no overlapping and complete overlapping, respectively

The secondary structures found in the agglomerated product can affect its properties and also the processing of IBs. Therefore, we assessed the secondary structure of the IBs by infrared (IR) spectroscopy. IR spectroscopy showed high similarity and overlaps in the IR spectra of all IBs indicating that the amount of correctly folded secondary structures were not significantly different (evaluated by degree of spectral overlap > 99.9%, (Schwaighofer et al. 2016)) independent of the induction strategy (exemplarily shown in Fig. 4 for 4% *q*_s,lac,max_ (small IBs, Ø = 408 nm); IPTG (medium IBs, Ø = 490 nm); and 57% *q*_s,lac,max_ (large IBs, Ø = 600 nm).

### IB processing

**Fig. 5:**
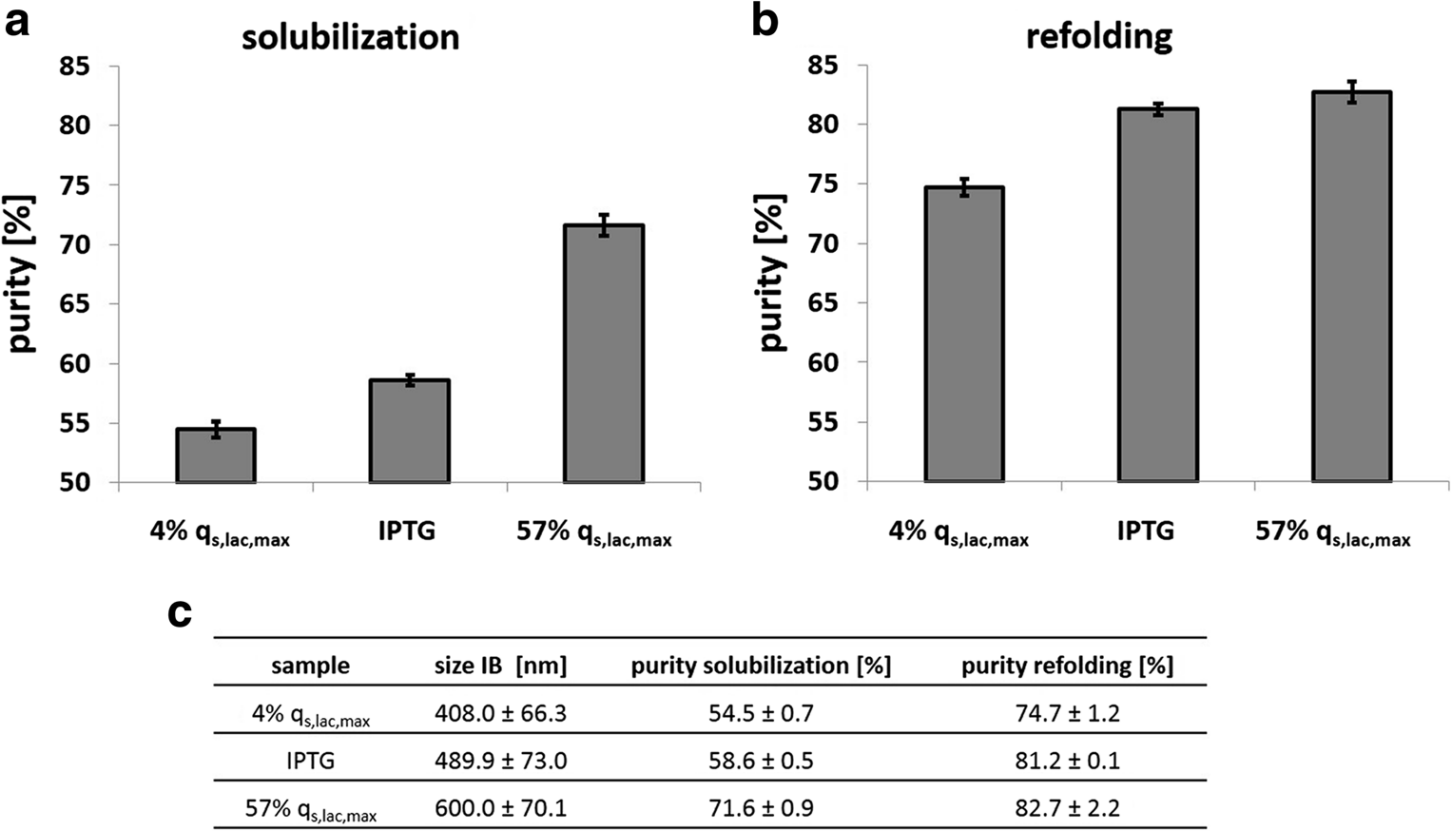
Impact of IB size on IB purity. **a** Purity determined by HPLC impurity monitoring using size exclusion chromatography (SEC) after solubilization with 2 M urea of IBs with a small (Ø = 408 nm), medium (Ø = 490 nm) and large (Ø = 600 nm) diameter. Standard deviation was evaluated from technical duplicates. **b** Purity of eGFP determined by HPLC impurity monitoring using SEC after refolding. Standard deviation was evaluated from technical duplicates. **c** Overview of results from solubilization and refolding with standard deviations

We hypothesized that the IB diameter and thus the surface area are crucial for subsequent IB processing, as (1) more impurities can adhere on particles with a larger surface area and (2) solubilization efficiency depends on accessibility to protein aggregates. To test the impact of the specific surface area (nm^2^/g_IB_) on IB processing and potentially omit necessary IB washing steps during production processes, we solubilized the different IBs with 2, 4, and 6 M urea, respectively, without any prior washing step. We found solubilization yields of > 99% for all IB preparations and all three urea concentrations. Since solubilizing at lower urea concentrations has the advantage of conserving correctly folded secondary structures resulting in an increased refolding yield (Margreiter et al. 2008; Singh et al. 2015b; Upadhyay et al. 2012), we used 2 M urea for solubilization of IBs to analyze IB purity. We used IBs from induction with IPTG and 4% *q*_s,lac,max_ and 57% *q*_s,lac,max_, respectively, to cover IBs of different sizes (Fig. 5). As shown in Fig. 5a, the purity of the IBs differed vastly. The high specific surface area of the small IBs formed at 4% *q*_s,lac,max_ caused the adherence of more impurities compared to the low specific surface area of large IBs formed at 57% *q*_s,lac,max_ (Fig. 5a, c). While small IBs showed a purity of only 55%, large IBs had a purity of more than 70%. Fig. 5b shows the purity after refolding, which was done by a standard dilution approach tailored for eGFP (Enoki et al. 2004). The purity of all IB preparations increased after refolding, as host cell derived impurities precipitated during this process step. After refolding, the purity was increased to 75% for small IBs, 81% for medium IBs, and 83% for large IBs. This observation confirms our hypothesis that a higher specific surface area attracts more impurities. The purity of IBs is of great importance as the presence of impurities can potentially reduce the refolding yield (Singh et al. 2015a). Furthermore, IB purity is a key aspect once IBs are directly used as nanomaterials and biomaterials (Diez-Gil et al. 2010; Garcia-Fruitos et al. 2009, 2012; Peternel and Komel 2011; Upadhyay et al. 2012; Villaverde et al. 2015).

Summarizing, we were able to show that tailored induction by lactose not only allows tuning of IBs size, but also IB purity. For the three different IB preparations we obtained a comparable refolding yield of > 95%. We expected these comparable values since we had found the same amount of correctly folded secondary structures in the different IBs by IR spectroscopy before (Fig. 4).

## Discussion

In this study, we showed that a mixed feed strategy with glucose and lactose not only impacts total product, soluble product, and IB titer in *E*. *coli*, but also IB properties, which in turn affects IB processing. Our method of tailored lactose induction allows precise tuning of the specific IB formation rate and is, thus, a valuable alternative to expression tuning by reducing the overall cell metabolism. Moreover, our approach allows prolonged production times and thus higher overall titers. Furthermore, it is of great interest that the size and the size distribution of IBs can be tuned by our method.

Size is an important property of IBs, since it significantly impacts IB harvesting and processing (Upadhyay et al. 2012). Furthermore, IB size is a crucial factor for potential direct applications of IBs as nanomaterials and biomaterials in regenerative medicine (Diez-Gil et al. 2010; Garcia-Fruitos et al. 2009, 2012; Peternel and Komel 2011; Upadhyay et al. 2012; Villaverde et al. 2015). We also showed that IB size correlates with purity and thus affects IB processing. We suggest to induce the cells at *q*_s,lac,max_ to obtain highest productivity and generate large IBs, which leads to a lower specific surface area and thus less adherent impurities. For eGFP IBs, we did not find any impact of induction on the amount of correctly folded secondary structures in the IBs. However, for more complex proteins, which often easily overwhelm the folding machinery, as well as for periplasmic proteins, where translocation is the rate limiting step, our strategy of tuning transcription by *q*_s,lac_ might be required to obtain higher product titers. Also, when expressing a protein which is toxic to *E*. *coli* and negatively affects its metabolism, it is beneficial to regulate recombinant protein expression to reduce metabolic burden and potential cell death. Summarizing, we present a method, which allows (1) tuning the specific formation rate of IBs, as well as (2) adjusting size, size distribution, and purity of IBs, which is not only fundamental for IB processing, but also for applications where IBs are directly used.

## Electronic supplementary material

ESM 1 (PDF 399 kb)
