# Supplementary material for: Teaching an old pET new tricks: tuning of inclusion body formation and properties by a mixed feed system in E. coli
Source: Appl Microbiol Biotechnol. 2017 Nov 20;102(2):667–76. doi: 10.1007/s00253-017-8641-6 (PMC5756567; doi:10.1007/s00253-017-8641-6)
Supplement: Supplementary file 1 — (PDF 399 kb) [file 253_2017_8641_MOESM1_ESM.pdf]

Applied Microbiology and Biotechnology

Supplementary Material for

Title: Teaching an old pET new tricks: Tuning of inclusion body formation and properties by a mixed feed system in *E.coli*

David J. Wurm[1], Julian Quehenberger[1,*], Julia Mildner[1,*], Britta Eggenreich[1], Christoph Slouka[1,2], Andreas Schwaighofer[3], Karin Wieland[3], Bernhard Lendl[3], Vignesh Rajamanickam[1,2], Christoph Herwig[1,2], Oliver Spadiut[1,§]

[1] Research Division Biochemical Engineering, Institute of Chemical, Environmental and Biological Engineering, TU Wien, Vienna, Austria

[2] Christian Doppler Laboratory for Mechanistic and Physiological Methods for Improved Bioprocesses, Institute of Chemical, Environmental and Biological Engineering, TU Wien, Vienna, Austria

[3] Institute of Chemical Technologies and Analytics, TU Wien, Vienna, Austria

*These authors contributed equally to this work

§ Corresponding author: Oliver Spadiut, TU Wien, Institute of Chemical, Environmental and Biological Engineering, Research Area Biochemical Engineering, Gumpendorfer Strasse 1a, 1060 Vienna, Austria. Tel: +43 1 58801 166473, Fax: +43 1 58801 166980, Email: oliver.spadiut@tuwien.ac.at

a

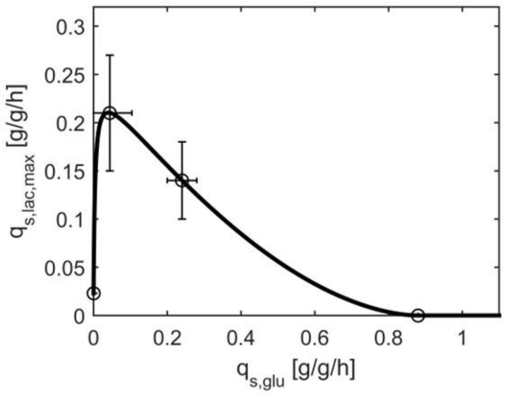

b

$q_{s,glu}$	$q_{s,lac,max}$
0.000 ± 0.000	0.023 ± 0.050
0.240 ± 0.040	0.140 ± 0.040
0.440 ± 0.004	0.210 ± 0.060
0.880 ± 0.050	0.000 ± 0.050

c

$$q_{s,lac} = q_{s,lac,max}{}^{*} \cdot \max\left(\left(1 - \frac{q_{s,glu}}{q_{s,glu,crit}}\right)^{n}, 0\right) \cdot \left(\frac{q_{s,glu}}{q_{s,glu} + K_A} + \frac{q_{s,lac,noglu}}{q_{s,lac,max}{}^{*}}\right)$$

$q_{s,lac}$	Specific lactose uptake rate [g/g/h]
$q_{s,lac,max}{}^{*}$	Maximum specific lactose uptake rate [g/g/h]
$q_{s,glu}$	Specific glucose uptake rate [g/g/h]
$q_{s,glu,crit}$	Critical specific glucose uptake rate up to which lactose is consumed [g/g/h]
$q_{s,lac,noglu}$	Specific lactose uptake rate at $q_{s,glu} = 0$ [g/g/h]
K_A	Affinity constant for the specific lactose uptake rate [g/g/h]
m, n	Type of inhibition (noncompetitive, uncompetitive, competitive)

d

$q_{s,lac,max}{}^{*}$	K_A	$q_{s,glu,crit}$	n	$q_{s,lac,noglu}$	NRMSE
[g/g/h]	[g/g/h]	[g/g/h]	[-]	[g/g/h]	[%]
0.23	0.0042	0.88	1.77	0.023	0.04

Supplementary Fig. S1. Maximum specific uptake rate of lactose ($q_{s,lac,max}$) as a function of the specific uptake rate of glucose ($q_{s,glu}$) for the BL21(DE3) strain expressing GFP. **(a)** Data points (open circles) were obtained from several batch and fed-batch cultivations and fitted by the mechanistic model displayed in (c). **(b)** Experimentally evaluated values of $q_{s,lac,max}$ at certain $q_{s,glu}$ for the BL21(DE3) strain expressing GFP. **(c)** Equation of mechanistic model describing $q_{s,lac,max}$ as a function of $q_{s,glu}$ **(d)** Fitted model parameters to the equation displayed in (c) for the BL21(DE3) strain expressing GFP, normalized root mean square error (NRMSE) indicates error between fitted curve and experimental data points. Data taken from our previous study (Wurm DJ, Veiter L, Ulonska S, Eggenreich B, Herwig C, Spadiut O (2016) The E. coli pET expression system revisited-mechanistic correlation between glucose and lactose uptake Appl Microbiol Biotechnol 100:8721-8729 doi:10.1007/s00253-016-7620-7)

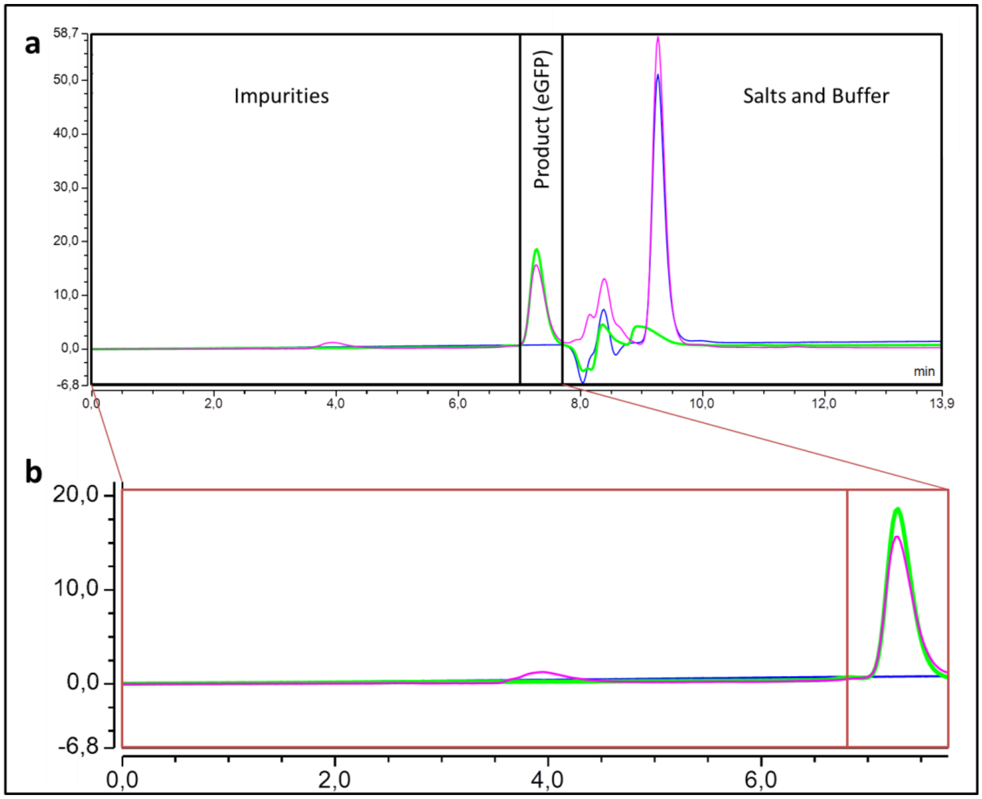

Supplementary Fig. S2. Exemplary Impurity monitoring data showing a Size Exclusion Chromatogram of solubilized and refolded product. Purity is evaluated by dividing product peak by total protein (Impurities + Product). Green, purified Green Fluorescent Protein. Blue, background (buffer). Pink, product sample. **a** whole chromatogram. **b** zoom of chromatogram.

Supplementary Table S1. Specific product titers of inclusion bodies **a**, soluble product **b** and total product **c**

a)

	Specific inclusion body titer					
induction time [h]	100% $q_{s,lac,max}$ [mg/g]	57% $q_{s,lac,max}$ [mg/g]	18% $q_{s,lac,max}$ [mg/g]	4% $q_{s,lac,max}$ [mg/g]	IPTG [mg/g]	no inducer [mg/g]
0	0.0 ± 0.0	0.0 ± 0.0	0.0 ± 0.0	0.0 ± 0.0	0.0 ± 0.0	0.0 ± 0.0
1	0.0 ± 0.0	0.0 ± 0.0	0.0 ± 0.0	0.0 ± 0.0	0.0 ± 0.0	0.0 ± 0.0
2	0.0 ± 0.0	0.0 ± 0.0	0.0 ± 0.0	0.0 ± 0.0	0.0 ± 0.0	0.0 ± 0.0
4	55.2 ± 6.2	31.7 ± 3.6	22.2 ± 2.5	26.2 ± 2.9	32.3 ± 3.6	0.0 ± 0.0
12	106.1 ± 11.9	97.0 ± 10.9	59.1 ± 6.6	41.4 ± 4.7	75.7 ± 8.5	0.0 ± 0.0

b)

	Specific soluble product titer					
induction time [h]	100% $q_{s,lac,max}$ [mg/g]	57% $q_{s,lac,max}$ [mg/g]	18% $q_{s,lac,max}$ [mg/g]	4% $q_{s,lac,max}$ [mg/g]	IPTG [mg/g]	no inducer [mg/g]
0	0.8 ± 0.1	3.4 ± 0.4	0.5 ± 0.1	7.6 ± 0.9	0.8 ± 0.1	0.0 ± 0.0
1	2.4 ± 0.3	5.3 ± 0.6	3.3 ± 0.4	4.9 ± 0.6	27.2 ± 3.1	0.0 ± 0.0
2	34.6 ± 4.0	20.6 ± 2.4	16.2 ± 1.9	12.1 ± 1.4	40.1 ± 4.6	0.0 ± 0.0
4	86.0 ± 9.9	55.7 ± 6.4	60.0 ± 6.9	30.7 ± 3.5	91.8 ± 10.6	0.0 ± 0.0
12	96.2 ± 11.1	142.0 ± 16.3	148.6 ± 17.1	175.4 ± 20.2	134.2 ± 15.4	0.0 ± 0.0

c)

	Specific total product titer					
induction time [h]	100% $q_{s,lac,max}$ [mg/g]	57% $q_{s,lac,max}$ [mg/g]	18% $q_{s,lac,max}$ [mg/g]	4% $q_{s,lac,max}$ [mg/g]	IPTG [mg/g]	no inducer [mg/g]
0	0.8 ± 0.1	3.4 ± 0.5	0.5 ± 0.1	7.6 ± 1.0	0.8 ± 0.1	0.0 ± 0.0
1	2.4 ± 0.3	5.3 ± 0.7	3.3 ± 0.5	4.9 ± 0.7	27.2 ± 3.7	0.0 ± 0.0
2	34.6 ± 4.7	20.6 ± 2.8	16.2 ± 2.2	12.1 ± 1.7	40.1 ± 5.5	0.0 ± 0.0
4	141.2 ± 19.3	87.4 ± 12.0	82.2 ± 11.3	56.9 ± 7.8	124.1 ± 17.0	0.0 ± 0.0
12	202.3 ± 27.7	239.0 ± 32.7	207.7 ± 28.5	216.7 ± 29.7	209.9 ± 28.8	0.0 ± 0.0

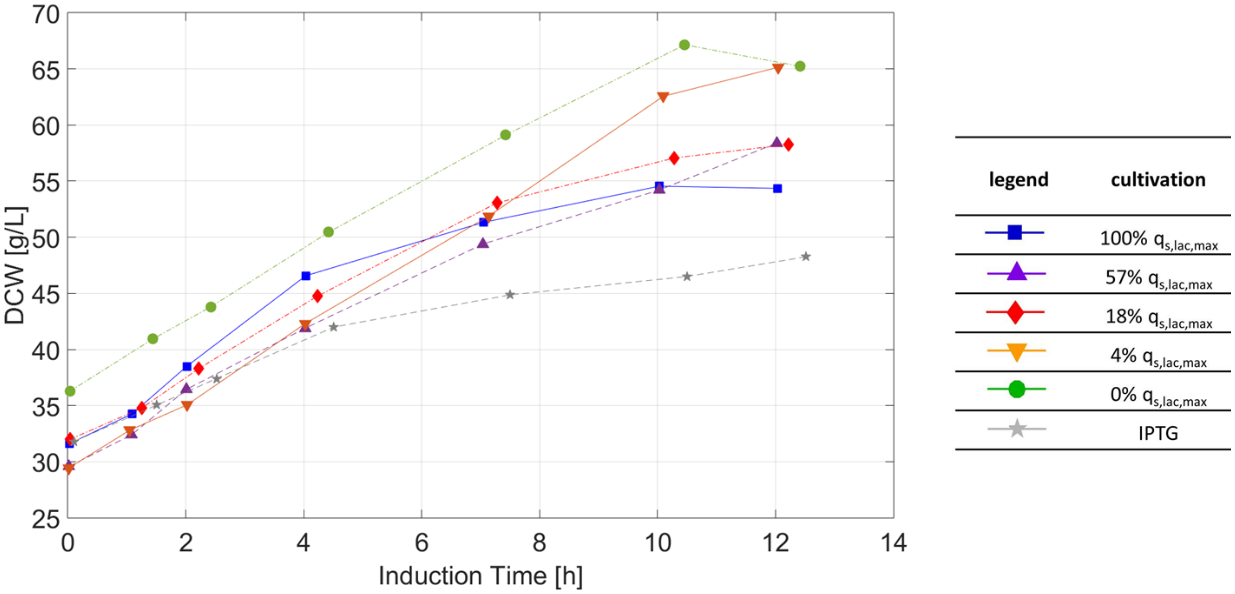

Supplementary Fig. S3. Dry cell weight concentration (DCW) as a function of induction time for all cultivations. Error of triplicates was below 3%.
